# Analysis of lysine acetylation in tomato spot wilt virus infection in *Nicotiana benthamiana*

**DOI:** 10.3389/fmicb.2023.1046163

**Published:** 2023-02-02

**Authors:** Yanwei Gong, Ying Li, Dongyang Liu, Lianqiang Jiang, Hui Liang, Yuanhua Wu, Fenglong Wang, Jinguang Yang

**Affiliations:** ^1^College of Plant Protection, Shenyang Agricultural University, Shenyang, China; ^2^Key Laboratory of Tobacco Pest Monitoring, Controlling and Integrated Management, Tobacco Research Institute of Chinese Academy of Agricultural Sciences, Qingdao, China; ^3^Liangshan State Company of Sichuan Province Tobacco Company, Mile, China

**Keywords:** lysine acetylation, TSWV, protein modifications, *Nicotiana benthamiana*, RBCL, post-translational

## Abstract

**Introduction:**

Kac is a model for all acylation modification studies. Kac plays a critical role in eukaryotes and prokaryotes. It is mainly involved in six major biological functions: gene expression, signal transduction, cell development, protein conversion, metabolism, and metabolite transport.

**Method:**

We investigated and compared the acetylation modification of proteins in healthy and tomato spot wilt virus (TSWV)-infected *Nicotiana benthamiana* leaves.

**Result:**

We identified 3,418 acetylated lysine sites on 1962 proteins acetylation of proteins in the TSWV-infected and control groups were compared; it was observed that 408 sites on 294 proteins were upregulated and 284 sites on 219 proteins (involved in pentose phosphate, photosynthesis, and carbon fixation in photosynthesis) were downregulated after the infection. Overall, 35 conserved motifs were identified, of which xxxkxxxxx_K_ Rxxxxxxxxx represented 1,334 (31.63%) enrichment motifs and was the most common combination. Bioinformatic analysis revealed that most of the proteins with Kac sites were located in the chloroplast and cytoplasm. They were involved in biological processes, such as cellular and metabolic processes.

**Discussion:**

In conclusion, our results revealed that Kac may participate in the regulation of TSWV infection in *N. benthamiana*.

## 1. Introduction

In the process of gene expression, functional gene products are synthesized using genetic information to ultimately regulate cell function. As a functional product, protein is a direct and key participant in all downstream biochemical pathways (Adams et al., 2009). From genome to proteome, biological complexity significantly increases because of post-translational modifications (PTMs). The central principle of biosynthesis is PTM of proteins. In plants, sexual reproduction, vegetative growth, and response to biotic and abiotic stresses are affected by functional changes caused by the folding or addition, or removal of functional groups on proteins (Ahlquist et al., 2003; Wang and Wang, 2019). More than 400 PTMs are known (Khoury et al., 2011). Glycosylation, ubiquitination, phosphorylation, and acetylation are the common types of PTMs, whereas some other types, such as adenosine acidification are rarely reported. Acetylation is the process of adding acetyl groups to molecules through acetyltransferase (Ahlquist et al., 2003). It is reported that many proteins can undergo acetylation modification, including histone (Wang et al., 2020), p53, and tubulin (Hammond et al., 2008). According to the various acetylation sites of proteins, acetylation can be divided into three types: Nα- acetylation, lysine acetylation, and O-acetylation (Diallo et al., 2019). In addition, protein acetylation is mainly involved in six biological functions: metabolism, protein conversion, histone/chromatin/gene expression, metabolite transport, signal transduction, and cell development cycle (Li et al., 2018).

The core histone of nucleosome is a small basic protein with conservative structure, and the tail of histone can protrude from the surface of nucleosome. Histone tails have various amino acid sites, which can exhibit completely different modifications (Phillips, 1963). Among them, histone acetylation modification was identified (Liu et al., 2021), and it was reported that it has a positive regulatory effect on transcription (Allfrey et al., 1964). Its physiological function was not identified until the last decade (Zhao and Garcia, 2015). It was reported that acetylation modification regulates histone and non-histone functions (Glozak et al., 2005; Liu et al., 2021). For example, p53 is the first reported non-histone protein with acetylation modification (Li et al., 2002), proving for the first time that Kac modification is involved in the regulation of non-histone activity. Several proteins with Kac are reported in rice, with 1,669 Kac sites on 1,024 proteins in vegetative cells and reproductive organs (Li et al., 2018) and 1,337 Kac sites on 716 proteins in the whole seedlings (Xiong et al., 2016). In addition, 2057 Kac sites were reported on 1,022 proteins in *Arabidopsis*, including many respiratory chain proteins and tricarboxylic acid cyclase (Hartl et al., 2017). With the detailed research, it was revealed that acetylation modification widely exists in eukaryotes and prokaryotes and in multiple components of cells, exhibiting diversity (Ouidir et al., 2015). It is involved in almost all biological processes, such as cell cycle regulation, cell morphology, cell metabolism, protein interaction, and enzyme activity. This modification is coordinated and regulated by histone/lysine acetyltransferases (hats/kats) and deacetylases (hdacs/kdacs). As an important means of protein function regulation, it plays an important role in defining protein structure and function (Zhang et al., 2013). However, most relevant studies are on rice and *Arabidopsis*, and only few studies have reported acetylation modification in plants from the Solanaceae family infected with tomato spot wilt virus (TSWV). Although some studies are available on Kac in plants, the effect of Kac on pathogen interactions, particularly inviral infection, and their regulation is not studied in plants (Ouidir et al., 2015; Hartl et al., 2017).

According to the classification report issued by the International Committee on Taxonomy of Viruses (ICVT), TSWV is a typical representative member of *orthotospovirus* in the *tospoviridae* family of *bunyavidales* (Zhang et al., 2013). TSWV is a newly defined genus, ranking second to TMV. The typical symptoms of this viral infection are concentric ring spots on leaves and fruits, dwarfing of some plants, distortion of young leaves, and yellowing of leaves, and long-term TSWV infection leads to the death of the whole plant (Pappu et al., 2009; Scholthof et al., 2011; Srinivasan et al., 2017). TSWV is a spherical particle wrapped in a double-layered lipid membrane with a thickness of approximately 5 mm. The diameter of the viral particle is approximately 80–120 nm. TSWV genome is composed of three single stranded genomic RNAs, which are divided into S (2,970 NT), M (4,767 NT), and L (8,913 NT) genomes according to the genomic size. Five viral proteins are expressed using negative or double sense coding strategy: L chain replicase protein RdRp (330 kDa) is responsible for regulating the replication of viral genomic RNA and the transcription of its mRNA; M-chain sense strand encodes a non-structural protein NSm (33 kDa), and the antisense encodes a glycoprotein precursor GP (127 kDa); Antisense strand of S genome encodes a non-structural protein NSs (52 kDa), and the sense strand encodes a nucleocapsid protein N (28 kDa; de Haan et al., 1991; Adkins et al., 1995; Chapman et al., 2003; Feng et al., 2020). In 2020, clones with TSWV infections were developed to study the interaction between TSWV and TSWV host (Rotenberg et al., 2015; Chen et al., 2019; Feng et al., 2020). However, to date, only a few host factors are reported that interact with TSWV coding proteins and participate in TSWV infection, and no PTMs of host proteins related to TSWV infection are reported.

In this study, using *N. benthamiana* as a model plant of Solanaceae, we investigated the changes in Kac in host proteins after TSWV infection. Overall, 4,570 peptides and 4,219 acetylated peptides were identified after LC–MS and bioinformatic analyses. This study laid a foundation for the further research on host defense in TSWV and breeding of disease-resistant species.

## 2. Experimental procedures

### 2.1. Tomato spot wilt virus inoculation and plant growth

As samples, 4-week-old *N. benthamiana* plants were selected and divided into two groups: control and treatment groups, with 3 plants in each group. The friction inoculation concentration of the treatment group was approximately 40×. The control group was treated with PBS buffer instead of TSWV. After inoculation, the plants were grown at 25°C under 16 h/8 h light/dark conditions for 7 days.

### 2.2. Protein extraction from *Nicotiana benthamiana*

First, 2 g leaves were collected and frozen in liquid nitrogen to retain the original composition of plant tissues. After removing the leaves form liquid nitrogen, they were quickly ground in a precooled mortar. The powder was placed in a 1.5 ml centrifuge tube containing Protease inhibitor lysate (CW2333, CW2200, CoWin Biotech, Beijing, China) and allowed to rest for 30 min at 4°C. After sufficient mixing, the suspension was centrifuged at 13,400 g at 4°Cfor 20 min. The supernatant was removed and stored at −20°C. Finally, the target proteins were dissolved in BCA working solution (prepared by mixing BCA and Cu reagent in 50: 1 ratio; it was sufficiently mixed till turbidity disappeared 37°Cfor 15–30 min (EC0001 Shandong Sparkjade Biotechnology Co., Ltd.). The absorption was measured at 562 nm using a photometer. The protein content was calculated according to the standard curve.

### 2.3. Database search

The Kac peptides were dissolved and separated using a reversed-phase analytical column (Acclaim PepMap RSLC C18 column, Thermo Fisher Scientific). The gradient phase composed of 2 to 10% solvent (0.1% formic acid in 98% acetonitrile) in 6 min, 10 to 20% in 45 min, 20 to 80% in 7 min and holding at 80% for at least 4 min, all at a flow rate of 250 nl/min on an UPLC system. The peptides were subjected to ESI/NSI sources followed by MS/MS in Q ExactiveTM Plus (Thermo Fisher Scientific) coupled online to UPLC. The Orbitrap was used to detect whole peptides and ion fragments at a resolution of 70,000 and 17,500, respectively, with NCE set at 30. The electrospray voltage was set at 2.0 kV. Automatic gain control (AGC) was used to prevent overfilling of the ion trap. The m/z range was from 350 to 1,800 for MS scans. The MS fixed first mass was set at 100 m/z and LC–MS/MS analyses were conducted at Micrometer Biotech Company (Hangzhou, China). The resulting raw data were processed using MaxQuant with integrated Andromeda search engine (v.1.5.2.8). Tandem mass spectra were searched against the same database. At the same time, the peptide was cleaved with trypsin. The number of peptide deletions was controlled to be less than 4, and each peptide had 5 modifications and 5 charges. The quality error of search was approximately 10 ppm; the main search error was approximately 5 ppm, and the error of fragment ions was approximately 0.02 Da. Further, the amino methylation modification of cysteine and oxidative modification of methionine were set as fixed modifications. For the acetylation of lysine and protein N-terminal, we set it as variable modification. We set the minimum peptide length to 7 bp. The false positive rate (FDR) threshold of proteins, modification sites, and peptides was set to 1%. All other parameter values in maxquant were set to default values. The location probability of the locus was set to >0.75.

### 2.4. Bioinformatic analysis

The acetylated proteins identified by Gene Ontology (GO)-RRB analysis were classified into three classes using Blast2GO software: molecular function, biological process, and cell component analysis. An adjusted *p* < 0.05 was considered significant. The subcellular localization of detected proteins was analyzed using the subcellular localization prediction software Wolfpsort, and the KEGG database was used to annotate protein pathways. The results of GO, KEGG pathway, and domain analysis were considered significant at *p* < 0.05. Functional analysis of protein domains was performed using the InterProScan software based on protein sequence alignment and the InterPro^1^ domain database. Motif-x software was used to analyze the amino acid composition of 21 amino acid regions in the upstream and downstream of acetylation sites of all acetylated proteins.

### 2.5. Protein–protein interaction network

For analyzing the interactions between proteins, we typically used the interaction gene/protein search tool (STRING) DATABASE43 and Cytoscape software (version 3.0.1) 44. When defining interaction confidence, the score was set to >0.7. All data were centrally presented only for the searched protein interaction. The dense junctions where interacting proteins appear were analyzed using a graph-theoretic clustering algorithm and a molecular complex detection (MCODE) plug-in toolkit.

### 2.6. Western blot analysis

The changes in the acetylated protein content after TSWV infection were detected with acetylated antibodies. The total protein extracted from plants was subjected to SDS-PAGE gel electrophoresis (precast protein plus gel, 12%, 10 wells, HEPES Tris, Yeasen, China). The separated proteins were transferred on the PVDF membrane (150 V, 40 min). The membrane was blocked with 5% BSA for 2 h and incubated with primary antibody (anti-Kac1:1500 PTM-801 PTM Biolabs, Hangzhou, China) overnight at 4°C (Actin 1:5000, ABclonal Biotechnology, Wuhan, China) and further with secondary antibody (goat anti-mouse IgG 1:10000, ABclonal Biotechnology, Wuhan, China) at room temperature for 3 h. The protein bands were visualized using Chemiluminescence imaging system (Tanon 5,200).

## 3. Results

### 3.1. Proteomic analysis of Kac peptides and proteins in tomato spot wilt virus-infected *Nicotiana benthamiana*

**Figure 1:**
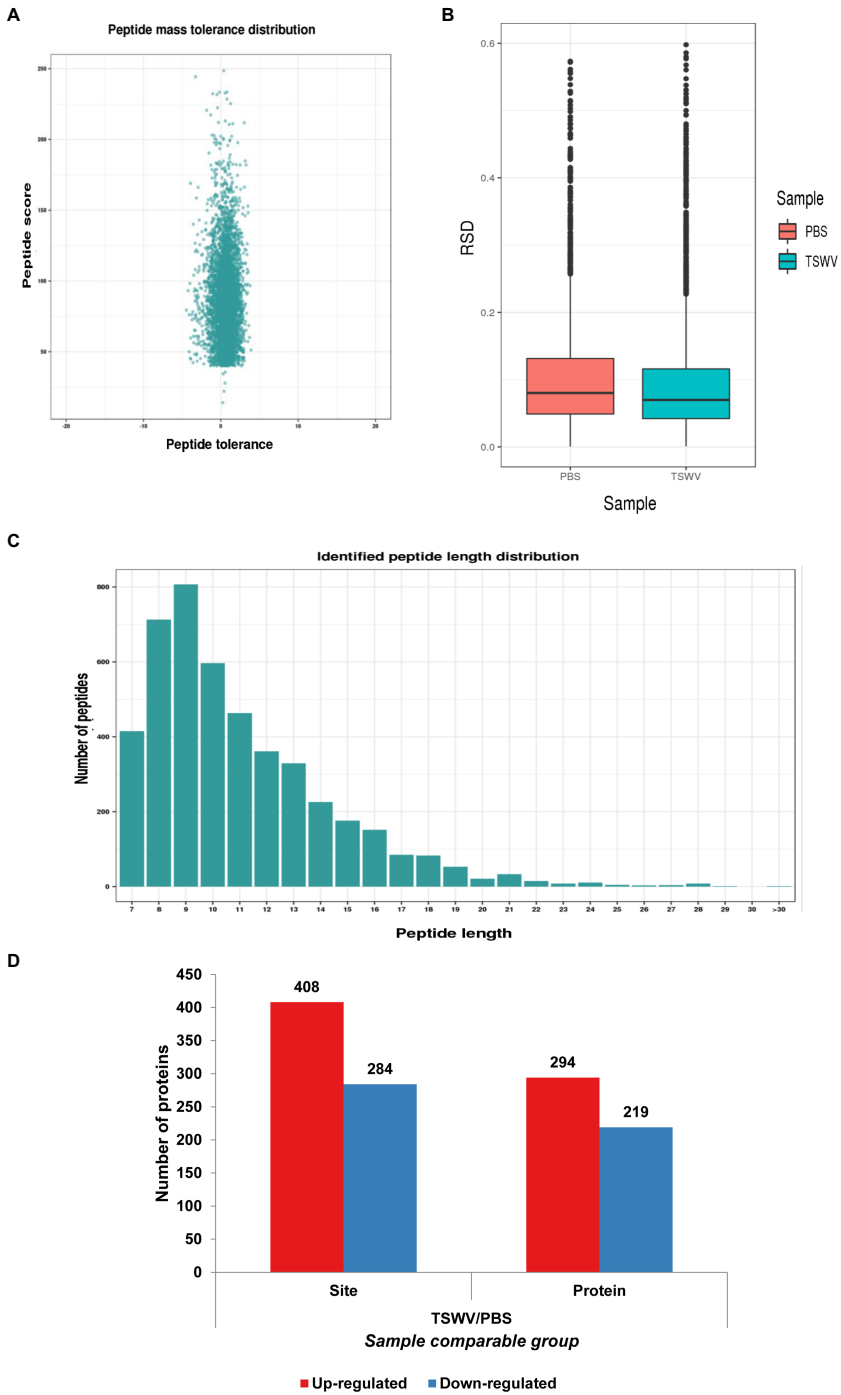
Protein acetylation in *Nicotiana benthamiana* after TSWV infection. **(A)** Photograph of *N. benthamiana* 7 days after inoculation with TSWV. **(B)** Coomassie Brilliant Blue was used to control the loading amount, and pan-anti-kac antibody against Kac protein was used for Western blot analysis. **(C)** Kac proteomic and modification processing. **(D)** LC–MS/MS spectra of acetyl peptides from downregulated proteins, namely, ribulose-bisphosphate carboxylase large chains and acetyl peptide DKLNK (1) YGR with one acetylation site at K164.

**Figure 2:**
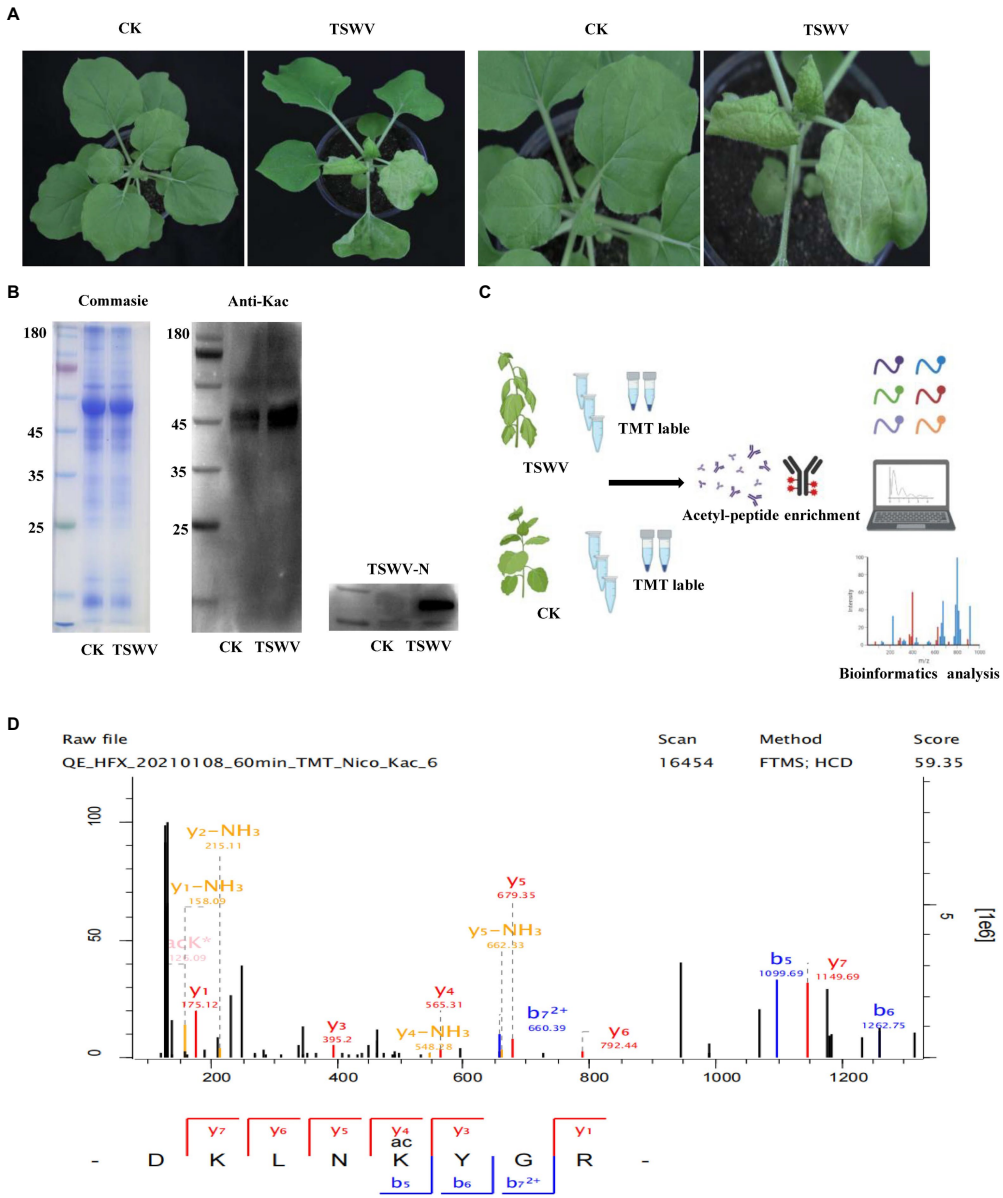
Analysis of Kac sites in *N. benthamiana*. **(A)** Quality error analysis of identified peptides. **(B)** Boxplot showing the sequence coverage of proteins that were quantified under each extraction condition. RSD, relative standard deviation efficient. **(C)** Distribution of peptide length. **(D)** The number of proteins and acetylation sites identified in the control and treatment groups.

Compared with the control, the viral content increased exponentially in the treatment group 7 days after inoculation with TSWV, and clear symptoms of the infection were exhibited (Figure 1A). The total proteins were extracted from the leaves of *N. benthamiana* after 7 days after of TSWV infection, and their content was determined using Coomassie Brilliant Blue. The amount of acetylated protein in the control and treatment groups was determined using western blotting with pan-acetylated antibody. The results revealed that the change in acetylated protein content in *N. benthamiana* leaves infected with TSWV was significantly higher than that in the control (Figure 1B). We performed LC–MS/MS analysis of lysine-acetylated peptides (Figure 1C), exemplified by spectral signals of acetylated peptides of downregulated proteins of the ribulose-bisphosphate carboxylase large chain (Figure 1D; Supplementary Table S1). To verify the validity and accuracy of the obtained data, the quality error of the acetylated peptide and repeatability of the sample were tested (Figures 2A,B). The length of the acetylated peptide segments was analyzed; the majority of the lengths focused on ranges from 7 to 19 (Figure 2C). In summary, 2,393 proteins were identified with 4,321 acetylated lysine sites after TSWV infection. The acetylated protein levels were compared between the control and treatment groups; 408 sites were upregulated on 294 proteins, and the 284 sites were downregulated in 219 proteins when considering a change threshold of at least 1.3-fold and a *p* < 0.05 in t-test (Figure 2D; Supplementary Table S2). Of all the proteins with altered acetylation levels, 19 were involved in the carbon-fixed degradation pathway, and the acetylation levels of 6 proteins were significantly reduced, with each protein having at least one acetylation site.

### 3.2. Pattern analysis of acetylation sites

**Figure 3:**
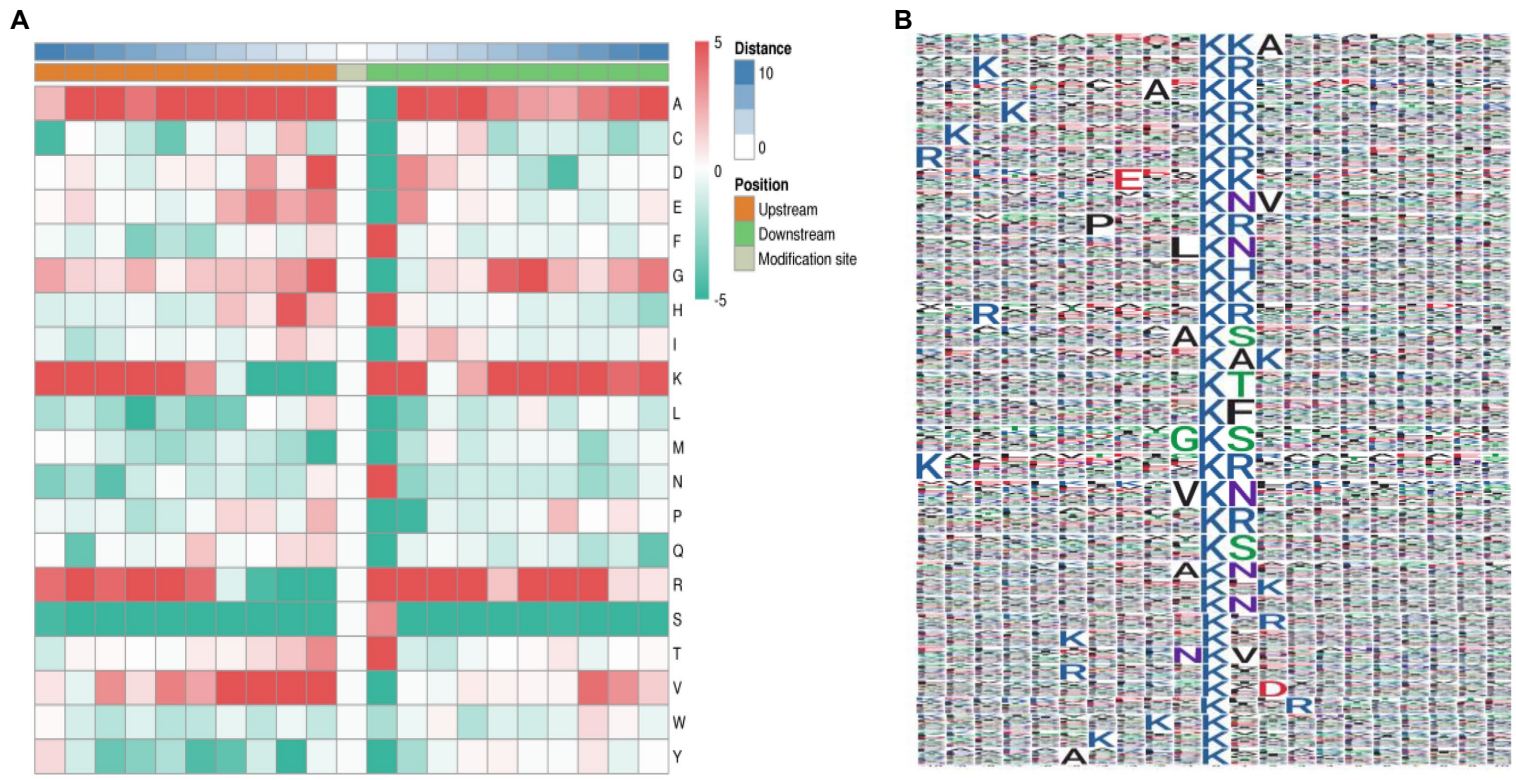
Characterization of lysine acetylation. **(A)** Heat map of the composition of common amino acids at acetylation sites. The middle white represents the Kac site, and the left and right represent the upstream and downstream amino acid residues based on Kac, respectively, with red representing tight and green representing loose. **(B)** Common acetylation motifs and conserved sites around the gene (the size of each letter corresponds to the frequency of amino acid residues at that site).

The characteristics and specific domains of acetylation sites were evaluated. To facilitate the future study on acetylation sites, The MOMO algorithm was set to motif-x, the minimum number of occurrences was set to 20, and the value of p threshold was 0.000001. Protein secondary structure analysis was performed using NetSurfP (Klausen et al., 2019). We used motif-x program to analyze the amino acid background of 10 to +10 on both sides of Kac site. A site-specific intensity map was generated to evaluate the significantly enriched amino acids around the acetylation sites. The concentration of amino acid frequency motifs revealed that the residues F, H, K, R, T, and N were preferentially selected at sites +1, +2, and +3, and residues A, D, G, and V were preferentially selected at sites −1, −2, and −3 (Figure 3A). However, serine residues were significantly lacking at all 20 sites around the acetylation sites. A total of 35 conservative motifs in K*A, K*R, A*K, K*K, E*K, K*NV, P*K*R, L*K*N, K*H, R*K*R, A*K*S, K*A, K*T, K*F, G*K*S, V*K*N, K*S, A*K*N, K*N, N*K*V, R*K, K*D, K*E and K*X were summarized from 4,219 acetylated peptides. Among these motifs, K*R represented 1,334 (31.63%) enriched motifs and was the most common combination (Figure 3B).

### 3.3. Functional distribution and subcellular localization of acetylated proteins in tomato spot wilt virus-infected *Nicotiana benthamiana*

To further classify the 1962 acetylated proteins in biological processes, cellular components, and molecular functions after TSWV infection in *N. benthamiana*, the GO analysis was performed. The results revealed that 212 acetylated proteins were involved in metabolism, 240 in cellular processes, and 141 in response to stimulation.

**Figure 4:**
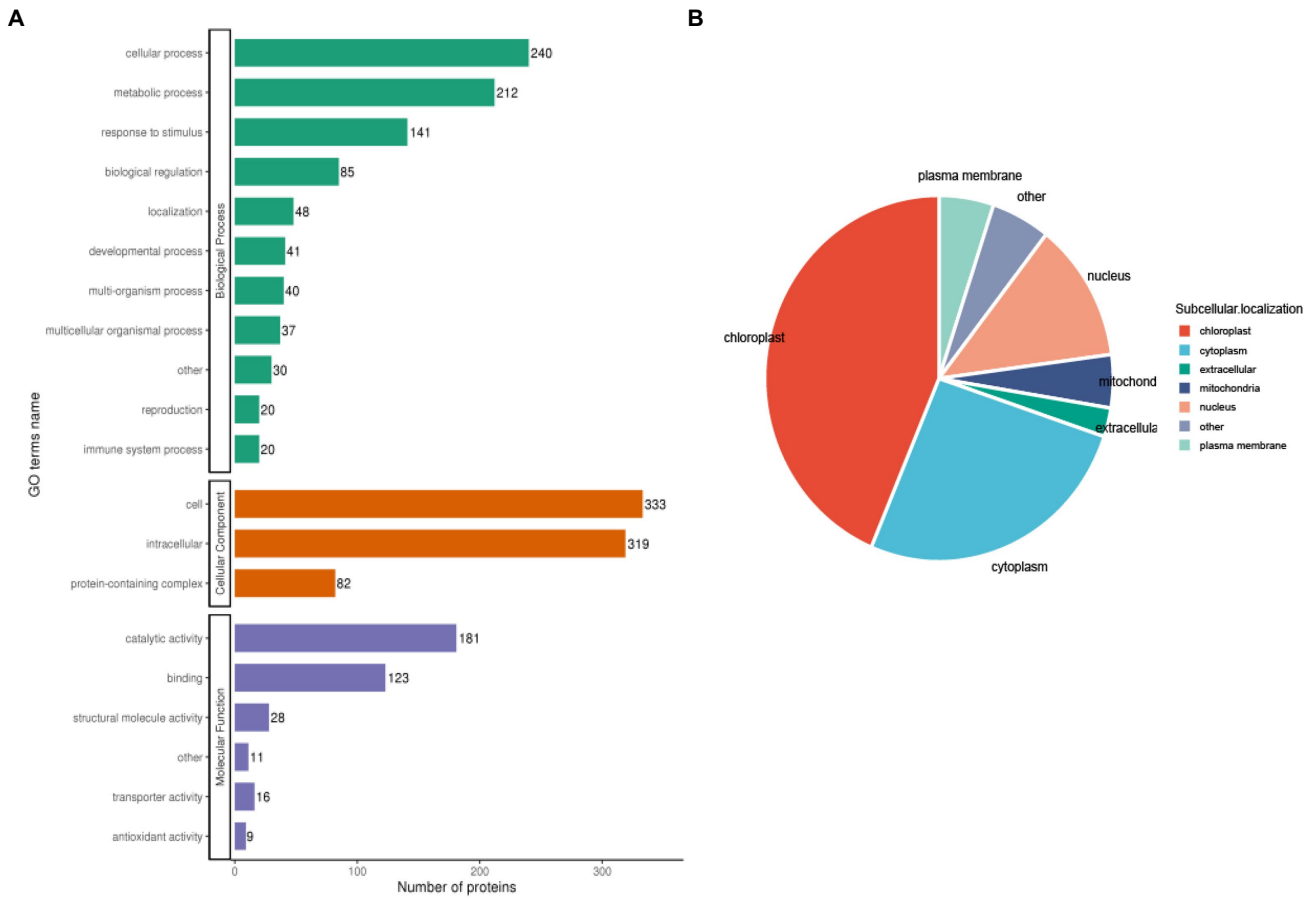
Functional distribution and subcellular localization of lysine-acetylated proteins in TSWV-infected *N. benthamiana*. **(A)** Quantitative profiles of lysine-acetylated proteins in biological processes, cellular components, and molecular functions. **(B)** The subcellular localization of lysine-acetylated protein is shown using a pie chart.

Moreover 333 and 319 acetylated proteins were in the cell and intracellular, respectively, and only 82 acetylated proteins were in the protein complex. Among the proteins from molecular functional class, most acetylated proteins were associated with catalytic activity (*n* = 181) or binding (*n* = 123; Figure 4A). The results indicated that the enzyme-metabolism-related proteins might have a high probability of acetylation in TSWV-infected *N. benthamiana.* In case of subcellular localization, the most acetylated proteins were located in the chloroplast in TSWV-infected *N. benthamiana* (Figure 4B). Some of the remaining acetylation proteins were mainly located in the cytoplasm, nucleus, and plasma membrane (Supplementary Table S3).

### 3.4. Enrichment analysis and domain enrichment of lysine-acetylated proteins in tomato spot wilt virus-infected *Nicotiana benthamiana*

**Figure 5:**
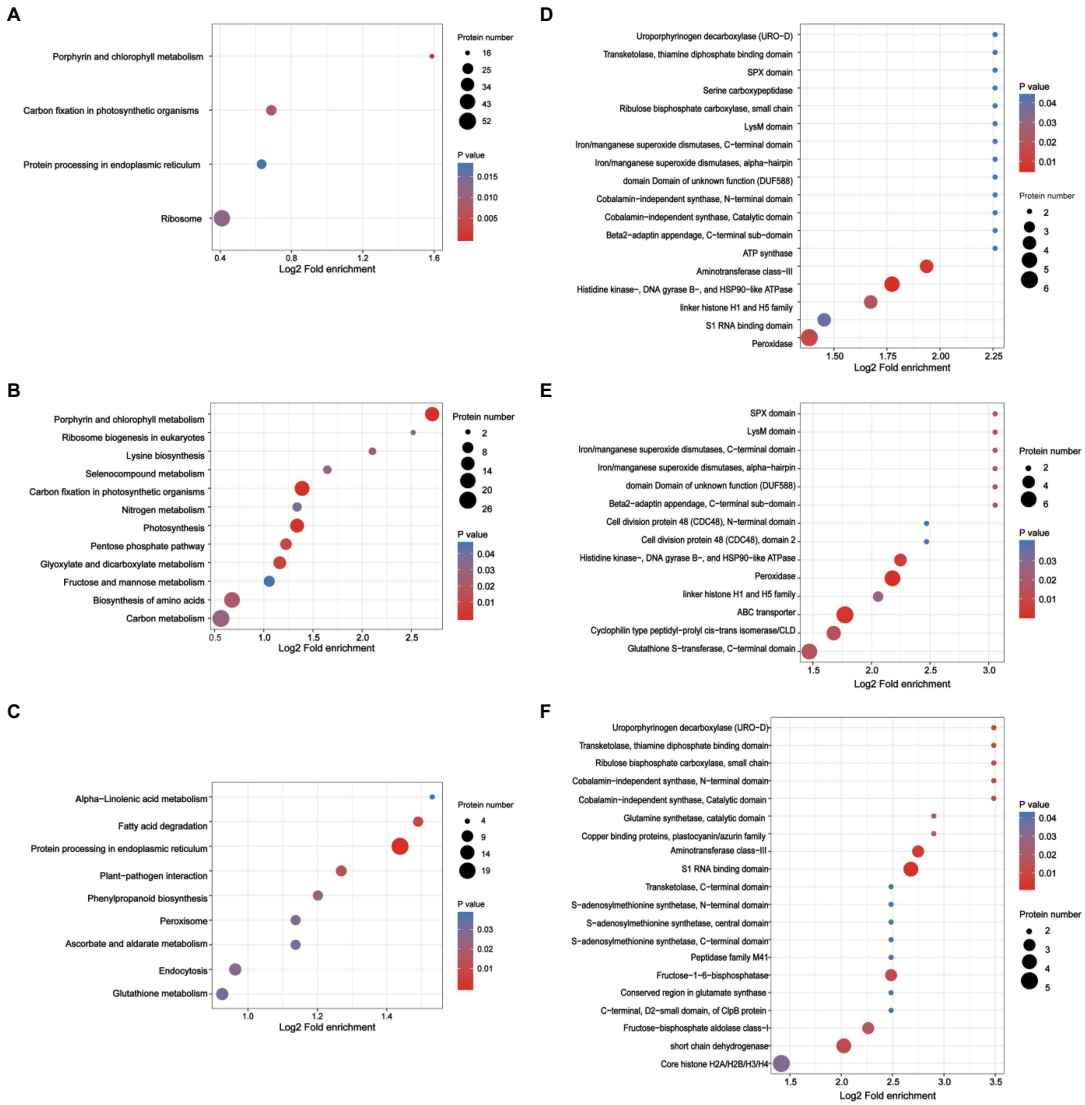
Analysis of the enrichment of lysine-acetylated proteins in *N. benthamiana* infected by TSWV. **(A)** KEGG pathway-based enrichment analysis of proteins in TSWV-infected versus uninfected plants (*p* < 0.05). **(B)** KEGG pathway-based enrichment analysis of upregulated proteins in TSWV-infected versus uninfected plants (*p* < 0.05). **(C)** KEGG pathway-based enrichment analysis of downregulated proteins in TSWV-infected versus uninfected plants (*p* < 0.05). **(D)** Concentration analysis of protein domains in TSWV-infected versus uninfected plants. **(E)** Protein domain enrichment analysis of upregulated proteins in TSWV-infected versus uninfected plants. **(F)** Downregulated proteins in TSWV-infected versus uninfected plants.

The response of acetylated proteins to TSWV infection in *N. benthamiana* was investigated based on KEGG analysis (Figure 5A). Downregulated acetylated proteins were mainly involved in the regulation of photosynthesis, carbon fixation, glyoxylic acid and dicarboxylic acid metabolism, and nitrogen metabolism (Figure 5B). Upregulated acetylated proteins were related to the regulation of biosynthesis of terpenoid skeletons, degradation of fatty acids, and protein processing in the endoplasmic reticulum (Figure 5C).

Kac was used as the preferred target for protein domain analysis using the InterPro domain database. The protein domain enrichment results revealed that 21 domains were enriched, which mainly included peroxidase, histidine kinase, DNA gyrase B, HSP90-like ATPase, and aminotransferase class III (Figure 5D). Meanwhile, the upregulated lysine-acetylated proteins included ABC transporter, peroxidase, glutathione S-transferase, and C-terminal domain cyclophilin-type peptidyl prolyl cis − trans isomerase/CLD domain (Figure 5E). Furthermore, downregulated lysine-acetylated proteins included core histone H2A/H2B/H3/H4, S1 RNA binding domain, short chain dehydrogenase, aminotransferase class III, and fructose-1,6-bisphosphatase (Figure 5F; Supplementary Table S4).

### 3.5. Analysis of the interaction network of acetylated proteins

**Figure 6:**
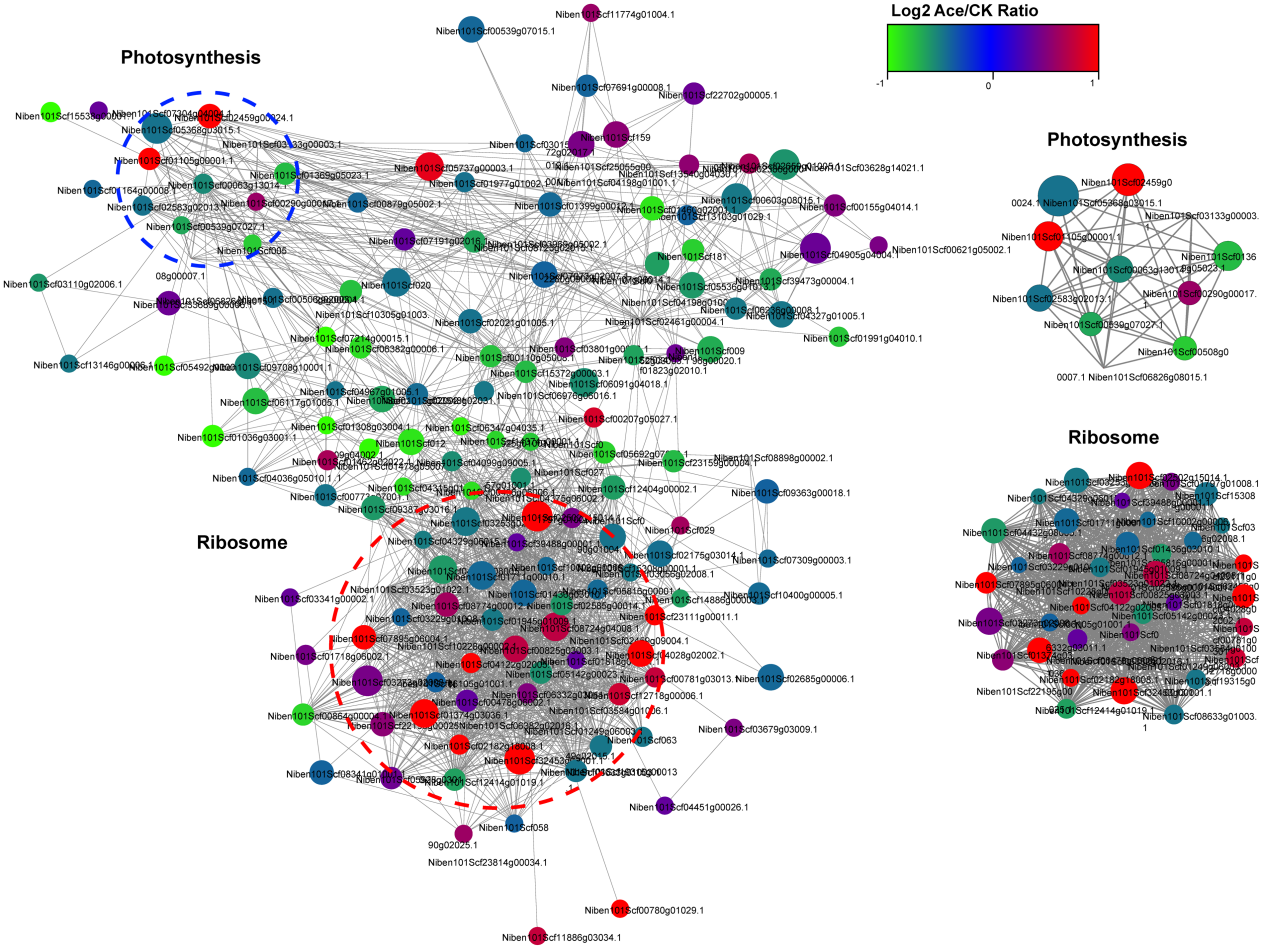
Interaction networks of differentially expressed acetylated proteins in TSWV-infected and healthy *N. benthamiana* plants.

To further investigate the role of Kac in TSWV infection in *N. benthamiana*, we visualized the protein–protein interaction network in a Cytoscape program using STRING database analysis. The results revealed that 155 acetylated proteins were mapped into the radiation map, and the network demonstrated how protein acetylation in various pathways was activated and mobilized in *N. benthamiana*. According to the Cytoscape program, 2 clusters of highly interconnected acetylated proteins were retrieved. The greater the density of mapping, the more the number of proteins they interacted with, indicating greater importance of proteins in the interaction network. The Internet network revealed that Kac was closely related to ribosomes and could regulate many metabolic pathways in the host (Figure 6; Supplementary Table S5).

### 3.6. Acetylated proteins involved in carbon fixation

**Figure 7:**
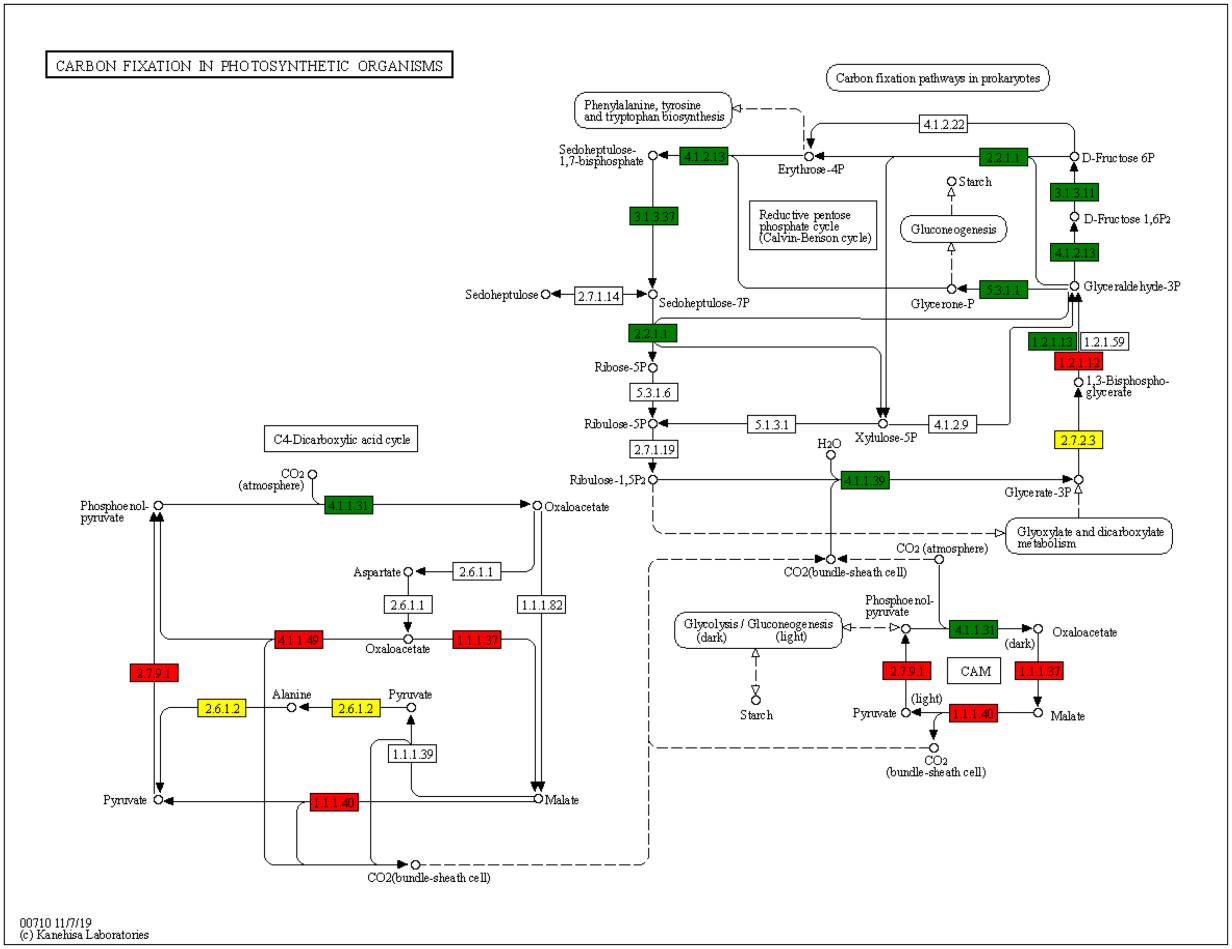
Analysis of metabolic pathways of carbon fixation. Red and green highlights are acetylated proteins that were significantly upregulated and downregulated, respectively, in the carbon fixation pathway in the treatment group compared with the control group. The yellow highlights indicate that at this point, acetylation level was upregulated and downregulated at the same time.

In KEGG pathway enrichment analysis, a large number of acetylated proteins related to carbon fixation pathway were identified. Further analysis revealed that the expression of acetylated GAPDH, PPDK, pckA, MDHI, and maeb was increased and that of acetylated. Downregulated proteins PPC, Aldo, glpx sebp, tkta, NbrbcL, FBP, TPI, and gapA (which play a role in the carbon fixation pathway of photosynthesis) was decreased in *N. benthamiana* infected with TSWV (Figure 7A). It is worth noting that the change in protein acetylation after TSWV infection was inconsistent with the change in protein expression. No change was observed in the expression of all upregulated proteins, and the change in acetylation may not change the protein content (Figure 7B).

### 3.7. Acetylation levels of NbrbcL protein were decreased in *Nicotiana benthamiana* after tomato spot wilt virus infection

**Figure 8:**
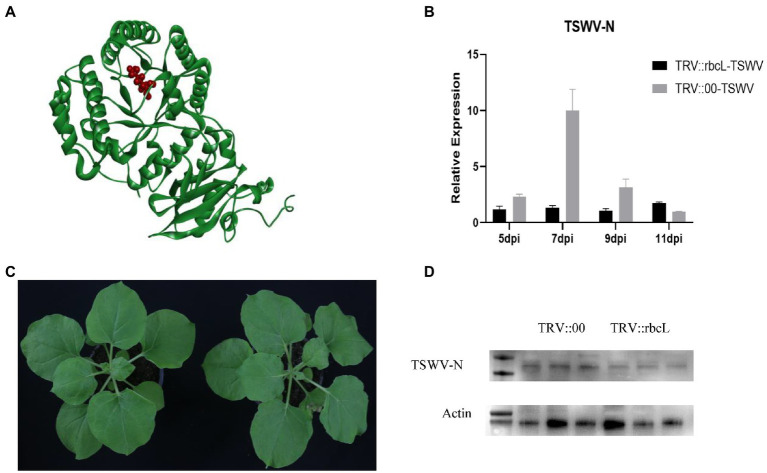
**(A)** Prediction of NbrbcL protein structure and acetylation site. Sites of acetylation are indicated in red. **(B)** Photographs were taken at 14 days after TRV infiltration. *TRV::00* is the control. **(C)** TSWV was inoculated after silencing *NbrbcL* 14 dpi, The RNA level of viral N protein was higher compared to the control *TRV::00* inoculated TSWV at the RNA level. **(D)** Samples inoculated with TSWV for 7 days were used to detect the differences in viral N expression using western blot analysis; 1–3 is TRV::00 inoculated with TSWV; 4–6 is TRV::NbrbcL inoculated with TSWV.

Comprehensive metabolic pathway analysis revealed that the acetylation level of NbrbcL protein in leaves infected with TSWV was lower than that in healthy leaves. The target protein structure and its acetylation site were predicted using PDB database^2^ (Figure 8A). Although acetylated protein bands were detected in both TSWV-infected and healthy leaves samples subjected to western blot analysis, the acetylation level of NbrbcL protein in TSWV-infected leaves was weaker than that in healthy leaves. To explore the relationship between *NbrbcL* gene and viral infection, The silencing efficiency was determined by comparing the expression levels of NbrbcL in *TRV*:: *NbrbcL* plants and *TRV*:: *00* control plants. No phenotypic difference was observed between *TRV*:: *NbrbcL* and control plants (Figure 8B). Next, *TRV*:: *NbrbcL* and control plants were infected with TSWV virus source, and viral infection was monitored for at least 1 week. The results revealed that the viral N gene expression in *TRV*:: *NbrbcL* was significantly lower than that in *TRV:: 00* control group from 5 to 11 days after viral inoculation (Figure 8C). Western blot analysis revealed that 7 days after the inoculation, the level of viral N protein in *TRV*:: *NbrbcL* was lower than that in *TRV::00* (Figure 8D). In conclusion, *NbNbrbcL* and its acetylation may play a role in TSWV infection in *N. benthamiana*.

## 4. Discussion

In 1927, it was reported that acetylation significantly affects protein specificity; however, no relevant large-scale study was performed till a long time (Allfrey et al., 1964). Kac ranks first in the acylation family and is the research model of all acylation modifications. To the best of our knowledge, this is the first study to classify the acetylated proteins in *N. benthamiana*, a model plant of Solanaceae family, infected with TSWV. Here, we detected 4,321 acetylated lysine sites on 2,393 proteins. The analysis of protein–protein interaction network revealed that these modified proteins in *N. benthamiana* not only participate in gene expression but also play an important role in various metabolic processes. The same modified proteins can participate in one metabolic pathway and perform multiple cellular processes at the same time; they may be positively correlated in one metabolism and negatively correlated in the other (Berger, 2007; Tribus et al., 2010; Bannister and Kouzarides, 2011; Li et al., 2015). This is sufficient to demonstrate that protein acetylation may be a common phenomenon in the process of host growth, reproduction, and resistance to external infection (Imai and Guarente, 2010; Kim et al., 2012; Lu et al., 2018). These results are consistent with previous studies on acetylated proteins in liver cancer (Hu et al., 2017), breast cancer (Guo et al., 2018), strawberry (Fang et al., 2015), and *Arabidopsis* (Konig et al., 2014).

The chloroplast organelles in the mesophyll cells of higher plants represent a sun-driven metabolic factory, performing photosynthesis and carbon fixation and ultimately providing fuel for life on our planet (Kirchhoff, 2019). In this study, a large number of acetylated proteins were located in the chloroplast, cytoplasm, and nucleus, revealing that viral infection may be closely related to acetylated proteins in the three organelles. Numerous studies have reported that chloroplasts can effectively activate defensive hormone response in the process of plant pathogen interaction, and viral proteins located on chloroplasts can promote viral pathogenesis (Medina-Puche et al., 2020). The carbon fixation reaction in photosynthesis starts from chloroplast matrix and ends in cytoplasmic matrix. A cyclic reaction, also known as Calvin cycle, continuously consumes ATP and NADPH and fixes CO_2_ to form glucose (Hirosawa et al., 2021). After the virus infects plants, it needs the help of the host to complete its own replication and proliferation. For example, when the plant defense response is activated, the C4 protein encoded by tomato yellow leaf curl virus relocates from the plasma membrane to the chloroplast, thus interfering with the biosynthesis of chloroplast-dependent antiviral salicylic acid (Wang et al., 2010). Based on these findings, we speculated that protein acetylation in photosynthesis process may play an indispensable role in viral infection.

In *N. benthamiana* leaves infected with TSWV, expression of a large number of acetylated proteins related to carbon fixation was altered, including GAPDH, PPDK, pckA, NbrbcL, FBP, TPI, and gapA. In the carbon fixation stage, green leaves absorb carbon dioxide from the environment through the pores, which cannot be directly reduced by reducing hydrogen. It must first bind to C5 (ribulose diphosphate) in plants. This process is called carbon dioxide fixation. After a carbon dioxide molecule is fixed by a C5 molecule, two C3 (12 glyceraldehyde-3-phosphate) molecules are quickly formed. Under the catalysis of relevant enzymes, C3 receives the energy released by ATP and is reduced by reducing hydrogen. NbrbcL plays an important role in this process, and the acetylation levels of NbrbcL in TSWV-infected and healthy plants were different. Therefore, we speculated that acetylated proteins may participate in carbon fixation in photosynthesis by affecting NbrbcL.

In summary, a large number of histone and non-histone lysine residues located in chloroplasts, cytoplasm, and nucleus are acetylated (Xu et al., 2021; Shvedunova and Akhtar, 2022). Some of these carbon fixation proteins may be related to viral infection and energy metabolism (Johnson, 2016). In this study, *N. benthamiana* infected with TSWV was used as the model, and acetylated proteins and their acetylation sites in it were revealed. This study broadened our understanding of Kac regulating the metabolic process and functional application in TSWV-infected *N. benthamiana*. This study on *N. benthamiana*, as a representative of plants from Solanaceae family, provided a basis for the future research on protein acetylation in plants from Solanaceae family infectec with viruses.

## 5. Conclusion

To the best of our knowledge, this is the first study assessing the Kac in TSWV-infected *N. benthamiana* and providing a resource for further exploration of the potential functions of Kac in the plant infections with segmented plants negative-stranded RNA viruses. The findings of this study provided insights into the function of Kac in in *N. benthamian* after sensing TSWV stages. However,further studies are needed to explore the detailed mechanism.

## Data availability statement

The datasets presented in this study can be found in online repositories. The names of the repository/repositories and accession number(s) can be found in the article/Supplementary material.

## Author contributions

JY and FW: conceptualization. DL: methodology. LJ: software. YG and YL: validation, formal analysis, writing-original draft preparation, and writing-review and editing. HL: investigation. YG: resources, data curation, and visualization. FW: supervision. JY: project administration and funding acquisition. All authors have read and agreed to the published version of the manuscript.

## Funding

This research was funded by Shandong Provincial Natural Science Foundation Project, grant number ZR202103070049 and China National Tobacco Corporation Green Tobacco Prevention and Control Major Special Projects, grant number 110202001033(LS-02)110202101045(LS-05)110202101027(LS-02).

## Conflict of interest

DL, LJ, and HL were employed by company Liangshan State Company of Sichuan Province Tobacco Company.

The remaining authors declare that the research was conducted in the absence of any commercial or financial relationships that could be construed as a potential conflict of interest.

## Publisher’s note

All claims expressed in this article are solely those of the authors and do not necessarily represent those of their affiliated organizations, or those of the publisher, the editors and the reviewers. Any product that may be evaluated in this article, or claim that may be made by its manufacturer, is not guaranteed or endorsed by the publisher.

## Footnotes

^1^\http://www.ebi.ac.uk/InterPro/

^2^\http://zhanglab.ccmb.med.umich.edu/I-TASSER/
